# Maternal Protein Restriction in Rats Alters the Expression of Genes Involved in Mitochondrial Metabolism and Epitranscriptomics in Fetal Hypothalamus

**DOI:** 10.3390/nu12051464

**Published:** 2020-05-19

**Authors:** Morgane Frapin, Simon Guignard, Dimitri Meistermann, Isabelle Grit, Valentine S. Moullé, Vincent Paillé, Patricia Parnet, Valérie Amarger

**Affiliations:** 1 Nantes Université, INRAE, IMAD, CRNH-O, UMR 1280, PhAN, F-44000 Nantes, France; morgane.frapin@univ-nantes.fr (M.F.); simon.guignard@inserm.fr (S.G.); isabelle.grit@univ-nantes.fr (I.G.); valentine.moulle@inrae.fr (V.S.M.); vincent.paille@univ-nantes.fr (V.P.); patricia.parnet@univ-nantes.fr (P.P.); 2 Nantes Université, INSERM, UMR 1064-CRTI, ITUN, F-44000 Nantes, France; dimitri.meistermann@univ-nantes.fr

**Keywords:** maternal nutrition, protein restriction, fetal brain, hypothalamus, differentiation, neurogenesis, transcriptomics, epitranscriptomics, mitochondria

## Abstract

Fetal brain development is closely dependent on maternal nutrition and metabolic status. Maternal protein restriction (PR) is known to be associated with alterations in the structure and function of the hypothalamus, leading to impaired control of energy homeostasis and food intake. The objective of this study was to identify the cellular and molecular systems underlying these effects during fetal development. We combined a global transcriptomic analysis on the fetal hypothalamus from a rat model of maternal PR with in vitro neurosphere culture and cellular analyses. Several genes encoding proteins from the mitochondrial respiratory chain complexes were overexpressed in the PR group and mitochondrial metabolic activity in the fetal hypothalamus was altered. The level of the N6-methyladenosine epitranscriptomic mark was reduced in the PR fetuses, and the expression of several genes involved in the writing/erasing/reading of this mark was indeed altered, as well as genes encoding several RNA-binding proteins. Additionally, we observed a higher number of neuronal-committed progenitors at embryonic day 17 (E17) in the PR fetuses. Together, these data strongly suggest a metabolic adaptation to the amino acid shortage, combined with the post-transcriptional control of protein expression, which might reflect alterations in the control of the timing of neuronal progenitor differentiation.

## 1. Introduction

The impact of altered nutrient availability on brain development during the perinatal period is widely acknowledged and is corroborated by both observations on humans and animal studies. Fetal malnutrition may be the consequence of an imbalanced maternal nutrition or placenta deficiency [1,2], with both possibly resulting in Intra Uterine Growth Restriction (IUGR) and a risk of preterm birth. In addition, very preterm infants often experience poor early postnatal growth, characterized by a deficit of lean mass [3]. This is often associated with neurological impairments during infancy [3,4]. Poor fetal growth is also known to confer a risk of developing metabolic diseases in adulthood according to the thrifty phenotype hypothesis [5], with the consequence being the impaired control of energy homeostasis. The hypothalamus, because of its central role in the regulation of energy homeostasis and food intake, has been intensely studied using animal models. Malnutrition in the perinatal period is associated with impaired hypothalamus development as well as altered leptin and insulin signaling, leading to defects in the control of food intake [6,7,8,9]. However, although the impact of perinatal nutrition on postnatal development of the hypothalamus and its functional consequences have been widely described [7,10], little is known about alterations that may take place during the morphogenesis of the fetal hypothalamus, probably because of the enormous complexity of the anatomy and functionality characterizing this brain region [11].

The development of the hypothalamus starts during early embryonic development with anterior–posterior patterning of the developing neural tube. The numerous nuclei of the hypothalamus are generated between E11 and E17 embryonic days in rodents [11,12]. This morphogenesis step is followed after birth, during the first two weeks of life in rodents, by the organization of nuclei that connect to each other and towards other regions of the brain [13].

The different cell types found in the hypothalamic nuclei originate from undifferentiated dividing neural progenitor cells (NPCs) residing in the ventricular zone of the brain, a transient embryonic layer of tissue. These NPCs, also named radial glial (RG) cells, are derived from neuroepithelial cells and give rise to committed neuronal and glial progenitors that migrate and differentiate according to a strictly defined program. In rodents, hypothalamic neurogenesis occurs prenatally between E12 and E17 and precedes astrogenesis that takes place during the postnatal period [13,14]. Because cell number and neurogenesis are determined during the prenatal period, the fetal environment, including maternal nutrition and metabolic status, may impact these processes, as already demonstrated by several studies on hippocampal and cortical tissues (reviewed in [15]). For instance, fetal malnutrition was associated with an alteration in the level of neuronal proliferation, maintenance and apoptosis of hippocampic cells [16,17]. Maternal protein restriction has been shown to reduce the proliferation of neural stem cells and to influence progenitor cell fate during embryonic cortex development in mice, resulting in increased cortex thickness [18].

Neural stem cells show a spectacular plasticity in their capacity to differentiate into a variety of cell types. This confers to the developing brain a great adaptability but also a high sensitivity to external cues. Hypothalamic progenitor cells were shown to respond in vitro to environmental stimuli such as the neurotrophic hormones insulin and leptin [19] or the endocrine disruptor Bisphenol A [20].

However, even if the impact of the perinatal environment on hypothalamus development is now clearly established, the underlying mechanisms remain largely unexplored. The proliferation and differentiation of neural stem cells and progenitors is strongly based on the precise control of the expression of specific genes, including pluripotency genes and lineage-specific genes. The maintenance in an undifferentiated state or the commitment into neuronal or glial differentiation requires a complex interplay between external cues, transcription factors, DNA-binding proteins, epigenetic control of gene expression and possibly other, as yet uncharacterized, mechanisms [21]. Overall, evidence is growing that gene expression is finely regulated both at the transcriptional and translational level. Given all this complexity, it makes sense that environmental factors can act on many levels.

The objective of this study was to identify early determinants of the impact of maternal protein restriction on hypothalamic development and to characterize, at the molecular level, early indicators of an impaired development. We use a well-characterized rat model of maternal protein restriction during gestation, which was designed to mimic placental defects, often resulting in the altered transfer of amino acids between mother and child [22]. We have previously shown that protein deficiency during gestation and lactation results in alterations in the development of the hypothalamus, leading to defects in the control of food intake [8,9] and metabolic alterations during adulthood [23].

Our strategy was to analyze fetal hypothalami at E17, which approximately represents the end of neurogenesis in rats [11,12]. Using 3′ digital gene expression sequencing (DGE-seq) for differential gene expression analysis, pathway enrichment analysis and N6-methyladenosine (m6A) RNA methylation assay, we have sought to identify possible molecular targets of fetal undernutrition that underlie alterations of in neurogenesis process in the hypothalamus.

## 2. Materials and Methods

### 2.1. Animals

All experiments were carried out in accordance with current guidelines of the local animal welfare committee and were approved by the Animal Ethics Committee of Pays de La Loire under reference 2016112412253439/APAFIS 7768. Nulliparous female Sprague–Dawley rats were purchased from Janvier Labs (Le Genest Saint Isle, France) and delivered to our facilities at the age of 7/8 weeks. On arrival, rats were housed (two per cage) under controlled conditions (22 °C, 12 h/12 h dark/light cycle) with free access to a standard diet (A04, SAFE-diets, Augy, France). After one week of acclimation, the estrous cycle was determined by vaginal smears and female rats in early estrous were mated overnight with a male. The presence of spermatozoa was verified the next day through vaginal smears and, when positive, this day was considered embryonic day 0 (E0). Pregnant rats were housed individually and randomly assigned to two experimental groups receiving either a control (C) diet containing 20% protein or a protein-restricted (PR) diet containing 8% protein (UPAE, Jouy-en-Josas, France). A detailed composition of the diets was described in [24]. At embryonic day 17 (E17), dams were anesthetized with 4% isoflurane and fetuses were sampled by caesarian section. Fetuses and placentas were weighed and brains were rapidly removed and dissected under a binocular magnifier to collect the hypothalamus. Some hypothalami were snap frozen in liquid nitrogen and stored at −80 °C for transcriptomic and proteomic analysis, while others were collected in cold PBS containing 2% glucose for cell biology experiments.

### 2.2. In Vitro Culture of Neurospheres from E17 Hypothalamus

For each litter, we collected six hypothalami to prepare neurospheres. After one wash in 2 mL of sterile PBS containing 2% glucose, hypothalami were mechanically triturated in 1 mL of NeuroCult Basal Medium (STEMCELL Technologies Inc., Vancouver, BC, Canada) using a 1 mL micropipette until a single-cell suspension was obtained. The cell suspension was then filtered on a 40 µm cell strainer (Greiner Bio-one International GmBH, Kremsmünster, Austria) and centrifuged at 500× *g* for 5 min. Cell pellets were resuspended in NeuroCult Basal Medium and viable cells were counted on a hemocytometer after eosin staining. All cells from one hypothalamus were seeded in a T-12.5 cm^2^ tissue culture flask containing 5 mL of Complete NeuroCult ^TM^ Proliferation Medium and incubated at 37 °C and 5% CO_2_. The obtained neurospheres were passed after 3 days in vitro. Briefly, cell passages were done by centrifuging neurospheres at 90× *g* for 5 min and incubating pellets with 200 µL of Accutase (STEMCELL Technologies Inc.) followed by gentle trituration to obtain single-cell suspensions. Cells were washed in NeuroCult Basal Medium, centrifuged at 500× *g* for 5 min and resuspended in NeuroCult Basal Medium, Complete NeuroCult ^TM^ Proliferation Medium or Complete NeuroCult^TM^ Differentiation Medium, depending on the following experiment.

### 2.3. Proliferation Test Using BrdU

Cell proliferation was measured with a BrdU Cell Proliferation colorimetric ELISA Kit (ab126556, Abcam, Cambridge, UK) following the manufacturer’s manual. This experiment was performed on passaged cells following neurosphere culture. Briefly, 20,000 cells resuspended in 100 µL of Complete NeuroCult ^TM^ Proliferation Medium were seeded in coated 96-well plates, incubated with 20 µL of 1× BrdU Reagent at 37 °C and 5% CO_2_ for 24 h and fixed with the provided solution. Fixed cells were incubated with anti-BrdU primary antibody, horseradish peroxidase-conjugated secondary antibody and tetramethybenzidine (TMB) substrate and absorbance at 450 nm was measured using a Varioskan LUX (ThermoFisher Scientific, Waltham, MA, USA)

### 2.4. Immunocytochemistry

Immature and mature neurons, undifferentiated cells and proliferative cell proportions were determined by immunochemistry using anti-TUJ1 (1:1000; MMS-435P-100 Eurogentec, Liège, Belgium), anti-MAP2 (1:100; #4542 Cell Signaling Technology, Leiden, The Netherlands), anti-NES (1:250; ab92391, Abcam) and anti-Ki67 (1:250; ab66155, Abcam) antibodies, respectively. Immunocytochemistry was performed on cells obtained after hypothalamus dissociation and on neurosphere cells resuspended in NeuroCult Basal Medium. Cells were fixed on Lab-Tek™ II Chamber Slide™ System (154534, ThermoFisher Scientific) with PBS and 4% paraformaldehyde (PFA). Blocking was done with an incubation step with PBS, 3% Bovine Serum Albumin (BSA) and 0.2% Triton for 1 h at room temperature and primary antibodies were added overnight at 4 °C. After three washings with PBS, secondary antibodies: Alexa 647-conjugated donkey anti-mouse (1:1000; 715-605-150, Jackson, Cambridge, UK), Alexa 647- conjugated donkey anti-rabbit (1:1000; 711-606-152, Jackson, Cambridge, UK) and biotin-conjugated goat anti-rabbit (1:1000; A24541, ThermoFisher Scientific were added and incubated for 1 h. After three washes, streptavidin Alexa 568 (1:1000; s11226, Molecular Probes, Eugene, OR, USA) was added to the wells containing biotinylated secondary antibodies. Cells were then incubated for 5 min at room temperature with DAPI (1/10,000; D3571 Molecular probes) and washed. The chambers were removed and the slides were mounted in VECTASHIELD^®^ Vibrance™ Antifade Mounting Medium (Vector Laboratories, Burlingame, CA, USA). Pictures of each well were obtained using ×20 magnification on Zeiss Axio Imager.M2m microscope and positive cells for each marker were automatically counted with an ImageJ [25] script based on object detection using signal intensity.

### 2.5. Mitochondrial Membrane Potential Determination

For each litter, two hypothalami were collected in cold PBS and 2% glucose was used to determine mitochondrial membrane potential using MitoTracker Red CMXRos (ThermoFisher Scientific) staining. Cell dissociation and counting were performed as previously described for cell culture. About 20,000 viable cells were incubated 30 min with Mitotracker Red CMXRos (500 nM). Cells were then centrifuged at 700× *g* for 3 min and resuspended in PBS before the fluorescence was read in Varioskan LUX (ThermoFisher Scientific) (Ex 579 nm, Em 599 nm). The values of the fluorescence were normalized to the number of viable cells seeded.

### 2.6. Western Blotting

Proteins were extracted from E17 fetal hypothalami stored at −80 °C with lysis buffer containing Radio Immunoprecipitation Assay (RIPA) lysis buffer (EMD Millipore Corp, Burlington, MA, USA), protease inhibitor and phosphatase I and II inhibitor (Sigma-Aldrich, Saint-Louis, MO, USA). Lysis buffer was added to each sample then hypothalami were shredded (Precellys^®^ Ozyme, 2 × 15 s at 5000 rpm) and centrifuged at 5590× *g* for 5 min at 4 °C. Protein concentrations were measured with Pierce™ BCA Protein Assay Kit (ThermoFisher Scientific) and 25 µg of proteins per sample were used for Western Blot. For subsequent labelling with the CSDE1 antibody, proteins were denatured with a heating step at 95 °C for 5 min with Laemmli Sample Buffer (Bio-Rad, Hercules, CA, USA) whereas, for the OXPHOS antibody, proteins were not denatured as recommended by the manufacturer. Proteins from the extracts were separated on a 4%–15% precast polyacrylamide gel (Bio-Rad) then transferred onto nitrocellulose membrane with the Trans-Blot Turbo Transfer System (Bio-Rad). For the membranes that were subsequently labelled with the OXPHOS antibody, total proteins were stained using the Revert^TM^ 700 Total Protein Stain (LI-COR Biosciences, Lincoln, NE, USA) following the manufacturer recommendations. The total amount of proteins per sample was quantified on the Odyssey (LI-COR Biosciences) using Image Studio Ver 5.2 (LI-COR Biosciences) and the EMPIRIA Studio software Ver 1.2 (LI-COR Biosciences) and used for normalization. The Revert stain was removed from the membrane using the Revert Reversal Solution before the incubation with the antibody. Membranes were then blocked in TBST containing 5% dried fat-free milk and incubated overnight with primary antibodies anti-CSDE1 (1:1000; ab 201688,), anti-β-ACTIN (1:7,500; A5441, Sigma-Aldrich) or anti-OXPHOS cocktail (1:250; ab 110413, Abcam) then one hour with secondary antibodies goat anti-rabbit IgG DyLight 800 (1:10,000, SA5-10036, ThermoFisher Scientific), goat anti-mouse IgG DyLight 680 (1:10,000, 35519, ThermoFisher Scientific) and goat anti-mouse IgG Dylight 800 (1:10,000, SA5610176, ThermoFisher Scientific). Immunolabelling was then revealed on the Odyssey (LI-COR Biosciences) using Image Studio Lite Ver 5.2 (LI-COR Biosciences). For the experiment with the anti-CSDE1 antibody, normalization of the signal was performed using the anti-β-ACTIN antibody.

### 2.7. m6A RNA Methylation Assay

The total amount of m6A in total RNA was measured using the m6A RNA Methylation Assay Kit (Fluorometric) (ab 233491, Abcam), following the manufacturer manual. For each sample, 200 ng of total RNA from E17 hypothalamus preparation were used.

### 2.8. 3′DGE Library Preparation, Differential Gene Expression Analysis and Enrichment Analysis

Total RNA and DNA were extracted simultaneously from hypothalami stored at −80 °C using NucleoSpin^®^ RNA columns and RNA/DNA buffer set (Macherey–Nagel, Hoerdt, France) following the manufacturer’s manual. Transcriptomic analysis was performed using 3′DGE (Digital Gene Expression)-sequencing in accordance with [26]. Briefly, mRNA libraries were prepared from 10 ng of total RNA from 96 individuals (eight males and eight females from each experimental group collected at three different ages (E17, D0 and D130)). Poly(A) mRNA tails were tagged using universal primers, sample-specific barcodes and a unique molecular identifier (UMI) and cDNA synthesis was performed using template-switching reverse transcriptase. Samples were then pooled, amplified and fragmented using a transposon fragmentation method that enriches for 3′ ends of cDNA. Fragments ranging from 350–800 bp were selected and sequenced on an Illumina Hiseq 2500. Paired-end sequencing was performed using a Hiseq Rapid SBS kit v2 50 cycles (FC-402-4022) and a Hiseq Rapid PE Cluster kit v2 (PE-402-4022). The first read of 16 bp corresponds to the sample-specific barcode and the second read of 57 bp to the mRNA in the 5′ 3′ direction. Alignments were done on RefSeq rat mRNA sequences (Rn6) by using BWA (version 0.7.15-0). The number of unique UMI associated with each RefSeq gene was counted. Only genes with three or more reads per sample in at least four samples were kept in the count table. Normalization and differential gene expression analyses were processed using DESeq2 (version 1.24.0) [27] with a correction for sex effect. Data from the 96 samples were used for normalization and differential expression analysis was performed separately for each age. Functional enrichment was done using FGSEA [28,29] from Gene Ontology (GO) [30], Kyoto Encyclopedia of Genes and Genomes (KEGG) [31] and Reactome [32] databases. The datasets generated for this study can be found at the European Nucleotide Archive (ENA) under the accession PRJEB35794. 

### 2.9. Mitochondrial DNA Quantification Using qPCR

Mitochondrial DNA was quantified by quantitative PCR using primers designed against the mitochondrial *CytB* gene (Forward 5′-TTCCGCCCAATCACCCAAATC-3′, Reverse 5′-GCTGATGGAGGCTAGTTGGCC-3′) and normalized against the geometric mean of the amplification signal from two nuclear genes: *Gapdh* (Forward 5′-TTCAACGGCACAGTCAAGG-3′, Reverse (5′-CTCAGCACCAGCATCACC-3′) and *Zfx-ZFy* (5′-AAGCATATGAAGACCCACAG-3′, Reverse 5′-CTTCGGAATCCTTTCTTGCAG-3′). Ten ng of total DNA were amplified in a total volume of 15 µL using the iTaq™ Universal SYBR^®^Green Supermix (Biorad) and 0.25 µM of each primer following the manufacturer’s instructions, in a CFX Connect™ Real Time PCR Detection System (Biorad). A relative amount of mitochondrial DNA was quantified using the 2^−ΔΔCt^ method.

### 2.10. Statistics

Either a Mann–Whitney or t-test was used to evaluate differences between groups and a two-way analysis of variance (ANOVA) was used to additionally assess sex effects. For each experiment, the tests are indicated in Figure and Table legends. The statistical analyses were performed using R software (version 3.6).

## 3. Results

### 3.1. Fetal and Placenta Weight at E17 Did Not Differ between Control and PR Fetuses

The number of pups per litter and weight gain during gestation did not vary between the control and PR dams (Table 1). Fetal and placenta weight were recorded immediately after caesarian section. Females weighed about 5% less than males in both control and PR groups, but there was no difference between the two groups in both sexes (Table 1). Placenta weight and Placenta–Fetus Ratio (PFR) were not impacted by maternal nutrition and fetus sex.

### 3.2. Hypothalami from PR Fetuses Contained a Lower Number of Cells

**Figure 1:**
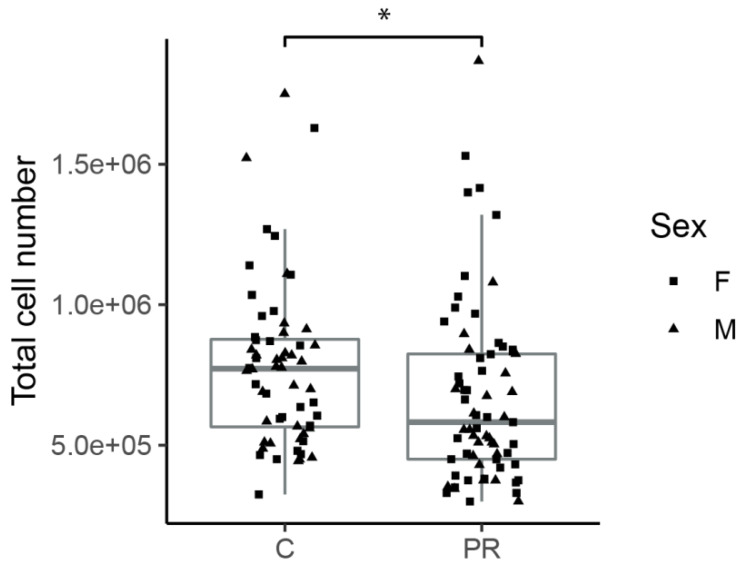
Total number of cells obtained from each E17 fetal hypothalamus after mechanical dissociation. The cells were counted on a hemocytometer (PR: *n* = 27 males and 42 females C: *n* = 32 males and 28 females). (two-way ANOVA: group effect * *p* = 0.04, sex effect *p* = 0.35) (boxplot: median, first and third quartiles).

The hypothalami from 129 fetuses (69 PR and 60 C) from 13 different litters were immediately dissociated into single cells. Cell number and viability were determined using a hematocytometer before subsequent analyses or cell culture. The percentage of viable cells was not different between both groups (control: 65.76 ± 10.39, PR: 68.39 ± 12.81, *t*-test *p* = 0.21). The total number of cells per hypothalamus was significantly lower in the PR group (6.7 ± 3.2 × 10^5^) compared to the control group (7.8 ± 2.9 × 10^5^) (two-way ANOVA group effect *p* = 0.04) (Figure 1). There was no difference between males and females.

### 3.3. The Proportion of the Several Cell Populations Present in E17 Hypothalami Did Not Differ between Control and PR Fetuses

**Figure 2:**
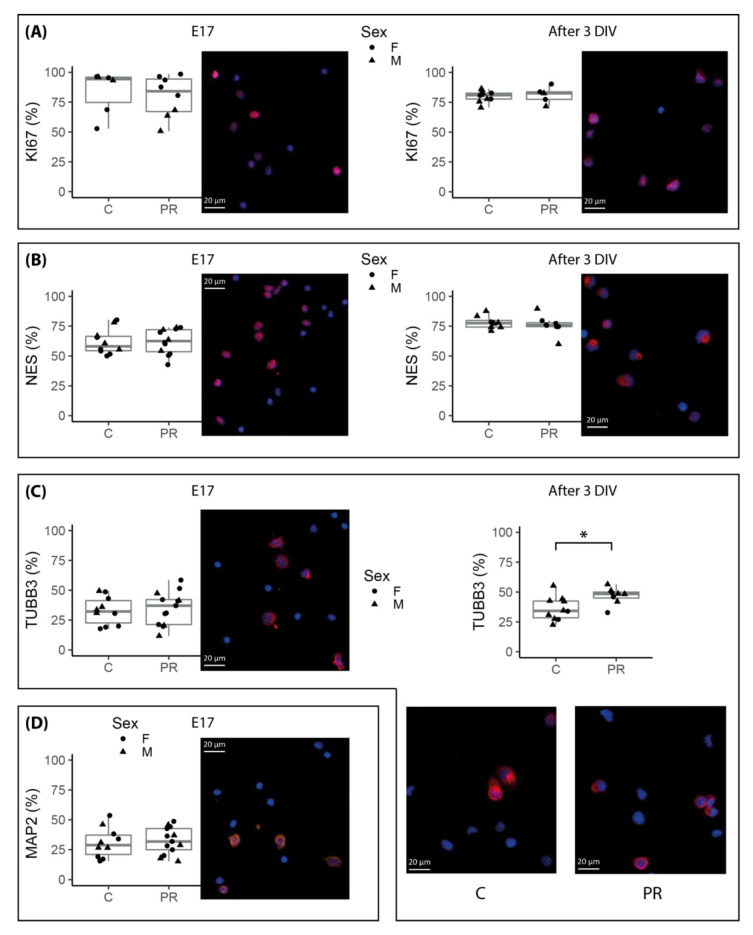
Neural cell type proportions in total hypothalamic cells sampled at E17 and after three days in vitro (3 DIV) labelled by immunocytochemistry and automatically counted on microscopy images. Each marker is illustrated by images obtained by optical microscopy (scale bars, 20 µm). Red and orange labelling is for markers of interest, Ki67 (**A**), Nestin (**B**), TUJ1 = TUBB3 (**C**) and MAP2 (**D**) and nucleus are labelled with DAPI in blue (*n* = 6–13 per group). Group and sex effects were tested using two-way ANOVA, * group effect *p* = 0.02. Non-significant *p* values are not mentioned. (boxplot: median, first and third quartiles).

In order to assess the impact of maternal diet on the proportion of the different cell types present in the hypothalamus at E17, dissociated cells were fixed on glass plates, stained with antibodies directed against Nestin, beta-III-tubulin, MAP2 and the proliferation marker KI67 and automatically counted after photomicrograph acquisition. Each cell type marker was counted on about 6000 cells per sample. The proportion of proliferating Ki67-positive cells was on average 82% in both groups. Nestin+ cells, representing undifferentiated NSCs and progenitors, accounted for about 60% of the cells (Figure 2). Beta-III-tubulin +cells, i.e., neural progenitors and young neurons accounted for 20% to 50% of the cells in both groups (Figure 2). Nestin and beta-III-tubulin markers were expressed at different levels in early or late progenitors but, based on this marker, we were not able to distinguish these two populations and therefore we could not accurately determine the level of these two types of progenitors. In contrast, more mature neurons, expressing MAP2, accounted for about 30% of the cells (Figure 2). Interestingly, although we observed a high inter-individual variability in the number of beta-III-tubulin+ and MAP2+ cells, the relative proportion of these two populations remained equal. We did not observe any significant difference between control and PR fetuses regarding the proportion of the different cell types, but a rather high inter-individual variability was observed that may alter the statistical power. This was especially true for Ki67+ cells, probably because Ki67 expression depends on the cellular cycle phase. Labelling experiments using a glial progenitor and astrocyte marker, GFAP, did not detect any positive cells at this early stage (data not shown).

### 3.4. Neurosphere Cultures from PR E17 Hypothalami Contained a Higher Number of Committed Neuronal Progenitors after Three Days of Proliferation In Vitro

For a subset of E17 hypothalami, dissociated cells were allowed to grow in vitro and form neurospheres. The purpose of neurosphere culture was to select and increase the number of cells that have the ability to proliferate, i.e., stem cells and progenitors. We deliberately limited the culture time to 3 days in order to minimize the biases introduced by a longer culture and preserve the initial intrinsic properties of the cells. After three days of culture, the proportion of proliferating Ki67+ cells was about 80% and rather homogeneous between samples (Figure 2). The number of Nestin+ cells was significantly higher in the neurospheres compared to the E17 hypothalami (76% versus 60% on average, *p* = 1.4 × 10^−5^), which was expected since neurosphere culture allows the selection of stem and progenitor cells. There was no difference between the control and PR fetuses regarding the proportion of Ki67+ and Nestin+ cells (Figure 2). A higher proportion of committed neuronal progenitors (beta-III-tubulin+) was present in the neurospheres coming from PR (47% on average) than control fetuses (36%) (two-way ANOVA group effect *p* = 0.02).

### 3.5. Neurospheres Cultures Did Not Show Any Proliferation Potential Difference between Control and PR Fetuses

Neurospheres were passaged after three days in culture and BrdU was added to the culture media for 24 h in order to test the cell proliferation capacities. There was no difference between groups in the amount of BrdU-incorporated (data not shown).

### 3.6. The Expression of More than 400 Genes Was Altered in PR E17 Fetal Hypothalamus

Because cell differentiation relies on the precise control of gene expression, we performed a global transcriptomic analysis using the digital gene expression sequencing (DGE-seq) approach. This technique is based on the sequencing of the 3′ end of mRNAs and is known to be highly sensitive for gene expression quantification but does not give any transcript-splicing information [26]. The experiment was performed on hypothalami of 16 individuals (eight males and eight females) from each experimental group collected at three different ages (E17, D0 and D130). However, because the objective of the present study was to evaluate the impact of maternal PR on fetal hypothalamus development, only the data from E17 fetuses will be presented here. An average of 3.4 million reads per sample was obtained and about 1.1 million reads with a single UMI were assigned to a known gene after alignment on the RefSeq transcript database. A total of 11,244 expressed transcripts were detected from the 18,946 transcripts from the RefSeq database (including the genes from the mitochondrial genome) (Supplementary Table S1). Among these, 6723 genes for which the normalized number of reads per E17 fetus was at least 10 were identified. The number of reads per gene varied from 10 to 21,000.

**Figure 3:**
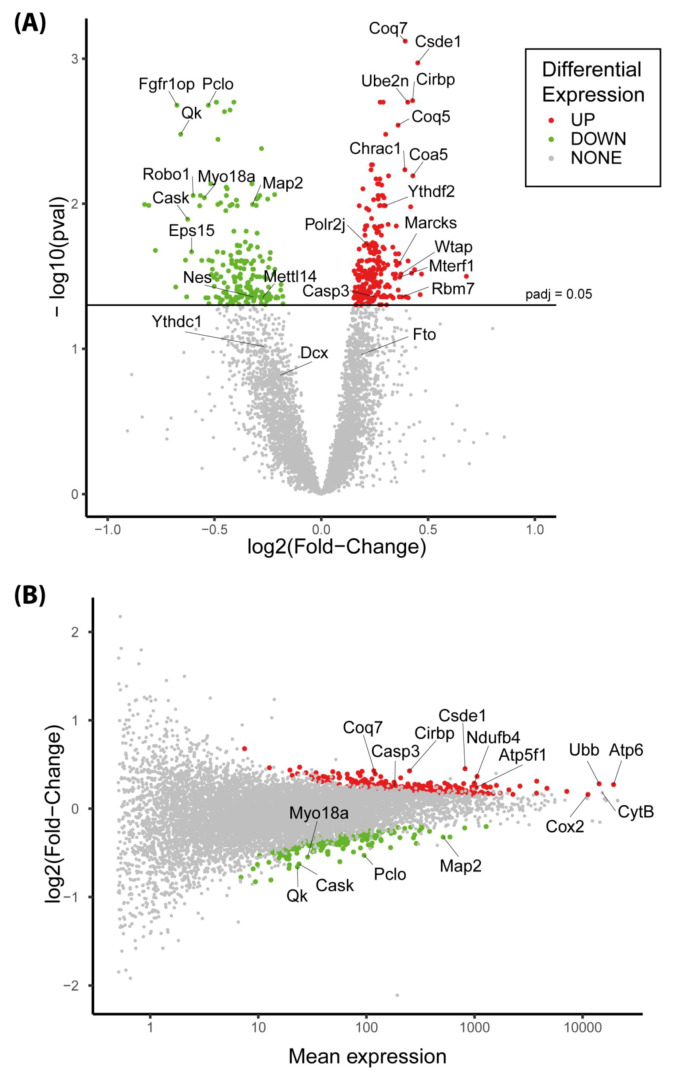
Volcano plot (**A**) and MA plot (**B**) of differentially expressed genes at E17 in the protein restriction (PR) group compared to Control group (PR: n = eight males + eight females, C: n = eight males + eight females). (padj = adjusted *p*-value from Deseq2 analysis). Mean expression corresponds to the average normalized read number.

A total of 440 genes (247 Up and 193 Down) were differentially expressed between the control and PR fetuses (adjusted *p*-value (padj) < 0.05) (Supplementary Table S1). Males and females were analyzed together and the data were corrected for sex effects. A volcano plot comparison showed that the magnitude of expression changes between both groups was relatively small, with log2 Fold Change (log2FC) ranging from −0.82 to 0.67, which means a fold change ranging from 0.56 to 1.6 (Figure 3). On the MA plot displaying the log2FC in relation to the level of expression, it appeared that the genes that were upregulated in the PR group had, on average, a higher expression level than genes that were downregulated (Figure 3). Among the 100 most-expressed genes (base mean > 1300 reads), 15 were upregulated in the PR group whereas none were downregulated. *Atp6*, *CytB* and *Cox2* were among the four genes with the highest expression rate, they are all encoded by the mitochondrial genome, involved in mitochondrial respiratory chain and were upregulated in the PR group (*Atp6*: log2FC = 0.2-padj = 0.04, *Cox2*: log2FC = 0.16-padj = 0.03 and *CytB*: log2FC = 0.18-padj = 0.09) (Figure 3) (Supplementary Table S1).

The top 20 up- and downregulated genes in the PR group compared to the control group are presented in Table A1 and Table A2 together with information regarding their function. Several genes potentially involved in the regulation of cell cycle, proliferation and cell migration were present in both lists (*Ube2n*, *Chrac1* and *Marcks* were upregulated, *Fgfr1op*, *Eps15*, *Robo1* and *Myo18a* were downregulated). Four genes encode RNA-binding proteins that are involved in RNA degradation, stability and translation (*Csde1*, *Cirbp*, *Rbm7* and *Qk*). In particular, *Csde1* and *Cirbp* genes, which play an essential role in the control of neuron differentiation, were highly expressed and upregulated in the PR group. Interestingly, several genes involved in the control of redox homeostasis and mitochondrial metabolism were also upregulated (*Coa5*, *Coq7*, *Mterf1*, *LOC100174910*).

**Figure 4:**
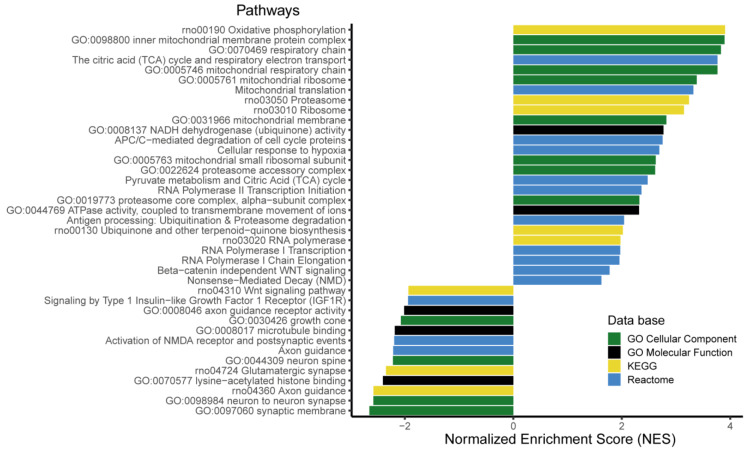
Selection of the gene families and metabolic pathways significantly enriched in differentially expressed genes (Fast Gene Set Enrichment Analysis (FGSEA), padj < 0.05). The pathways with a Normalized Enrichment Score (NES) > 0 are enriched in genes upregulated in the PR group and pathways with an NES < 0 are enriched in genes downregulated in the PR group.

A Fast Gene Set Enrichment Analysis (FGSEA) was performed in order to identify gene families and/or metabolic pathways significantly impacted by the maternal diet. Briefly, the principle of this method is to rank the genes according to the significance of their differential expression and fold change and to test which pathways are significantly enriched in genes that are globally up- and/or downregulated [33]. We used several databases (KEGG, Reactome, GO ) that are partly redundant but more or less complete in order to conduct an exhaustive search of metabolic pathways and cellular processes that may be impacted by the maternal diet. A total of 585 pathways were significantly enriched (enrichment adjusted *p*.value < 0.05) (Supplementary Table S2). The FGSEA analysis generates a Normalized Enrichment Score (NES) which reflects the degree to which a gene set is overrepresented at the extremes of the ranked list of genes [33]. A selection of the top pathways is presented on Figure 4. Pathways with an NES > 0 or NES < 0 contain genes that are predominantly over- or under-expressed, respectively, in the PR group compared to the control group.

A large number of pathways related to mitochondrial energy metabolism were significantly enriched with an NES > 0 (Figure 4, Supplementary Table S2). These pathways contain the genes encoding proteins of the mitochondrial respiratory chain complexes, including NADH dehydrogenase (*Ndufa1*, *Ndufa12*, *Ndufa13*, *Ndufa2*, *Ndufa4*, *Ndufa5*, *Ndufa7*, *Ndufab1*, *Ndufb4*, *Ndufb8*, *Ndufv2*), ATP synthase (*Atp5f1*, *Atp5f1c*, *Atp5mf, Atp5pf*, *Atp6*) and ubiquinone synthase (*Coq5*, *Coq7*). Genes encoding enzymes from the Tricarboxylic Acid (TCA) cycle and pyruvate metabolism were also significantly over expressed in the PR fetuses, as well as genes encoding mitochondrial ribosomal proteins (*Mrpl17*, *Mrpl27*, *Mrpl30*, *Mrpl35, Mrpl50*, *Mrpl51*, *Mrpl53*, *Mrps11*, *Mrps18c*) that are involved in mitochondrial translation.

Several pathways related to the proteasome complex were significantly enriched in upregulated genes (Figure 4). The *Ubb* gene, encoding the ubiquitin protein, which forms a polymerized chain that binds to target proteins and shuttles them to the proteasome, was also upregulated in the PR group. In addition, several genes encoding the subunits of the RNA polymerase II (*Polr2e*, *Polr2i*, *Polr2j*) and proteins associated with RNA transcription (*Ccnh*, *Taf6*, *Taf9*, *H3f3b*, *Tbp*) were over-expressed in the PR group, as illustrated by the enrichment of RNA polymerase-related pathways.

The enriched pathways with an NES < 0, i.e., containing genes that were downregulated in the PR group were predominantly related to neurogenesis, axonal growth and synaptogenesis (Figure 4), including the Wnt signaling pathway, a key element in neurodevelopment [34].

### 3.7. Mitochondrial Membrane Potential Was Enhanced in the Hypothalamus of E17 PR Fetuses

**Figure 5:**
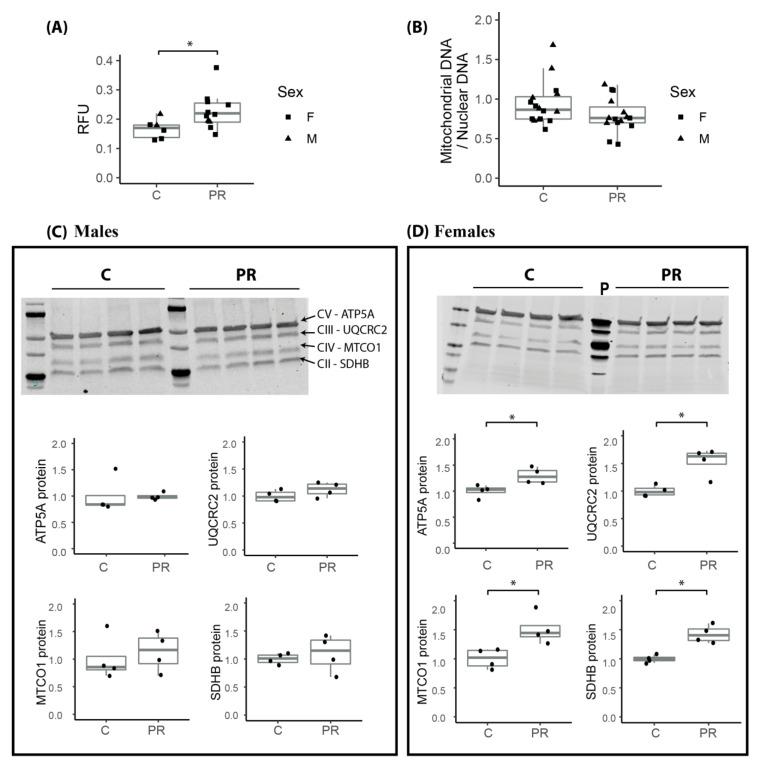
Mitochondrial membrane potential, DNA and protein quantification from total E17 fetal hypothalamus. (**A**) Mitochondrial membrane potential was quantified in dissociated total fetal hypothalamus cells by MitoTracker Red CMXRos and expressed in Relative Fluorescence Units (RFU)/lived cells x 1000 (C: *n* = two males + four females, PR: *n* = two males + nine females) (two-way ANOVA: group effect * *p* = 0.04, sex effect *p* = 0.91). (**B**) Mitochondrial DNA was quantified using qPCR amplification on total DNA from E17 whole hypothalamus of the mitochondrial *CytB* gene and normalized with two nuclear genes *Gapdh* and *Zfx-Zfy*. (**C**,**D**) Four proteins from the mitochondrial respiratory chain complexes (SDHB, MTCO1, UQCRC2 and ATP5A from Complexes II, III, IV and V, respectively) were quantified using Western blot on total proteins from E17 total hypothalamus in four males (**C**) and four females (**D**) from each group. Total protein stain was used for normalization. (Mann Whitney * *p* < 0.05) (p: Positive control = mitochondrial protein extract from rat heart tissue lysate (Abcam ab110341)) (boxplot: median, first and third quartiles).

In order to validate, at the cellular level, the relevance of the observed upregulation of several genes related to the mitochondrial oxidative phosphorylation metabolism, we quantified the mitochondrial membrane potential by using a MitoTracker staining method on total cells dissociated immediately after sampling the E17 fetal hypothalami. The MitoTracker molecules diffuse through the inner membrane of active mitochondria, in proportion to their membrane potential, making it possible to quantify the mitochondrial membrane potential. We observed a significantly higher mitochondrial membrane potential in the hypothalamus cells from the PR E17 fetuses (Figure 5).

Then, we quantified mitochondrial DNA in E17 fetal hypothalamus, in order to check if the increase in gene expression could be due to a higher number of mitochondrial genome copy numbers and therefore a higher number of mitochondria per cell (Figure 5B). There was no difference in the amount of mitochondrial DNA between the control and PR fetuses, which suggests that the difference in gene expression level were linked to mitochondrial activity rather than number.

We also quantified, at the protein level, four proteins, SDHB, UQCRC2, MTCO1 and ATP5A, from the complexes II, III, IV and V, of the mitochondrial respiratory chain, respectively (Figure 5C,D). The four proteins were significantly overexpressed in females from the PR group (Figure 5D) whereas there was no difference between control and PR males (Figure 5C).

### 3.8. The Csde1 Gene, a Major Regulator of Neuronal Differentiation, Was Upregulated in the PR Group

**Figure 6:**
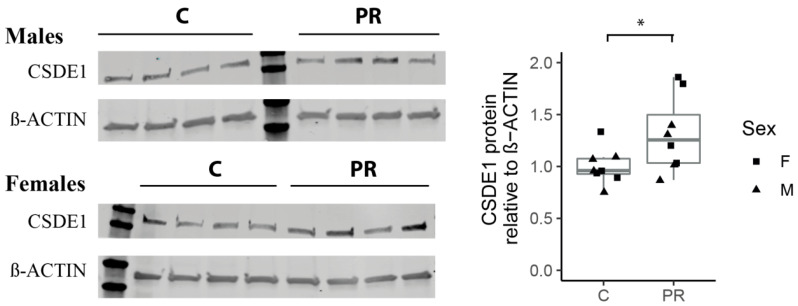
Quantification of the CSDE1 protein in E17 fetal Hypothalamus using Western blot. (PR: *n* = four males + four females, C: n = four males + four females) (ANOVA, group effect * *p* = 0.04, sex-effet *p* = 0.18) (boxplot: median, first and third quartiles).

Among the top 20 upregulated genes (Table A1), the *Csde1* gene encodes an RNA-binding protein implicated in the post-transcriptional regulation of a subset of cellular mRNA and was recently shown to prevent neural differentiation [35]. *Csde1* was highly expressed in the fetal hypothalamus (mean normalized read count = 817) and its expression level was about 35% higher in the PR group (log2FC = 0.45, padj = 0.001). Since CSDE1 protein is known to regulate the translation of its own mRNA, the amount of transcript does not necessarily reflect the amount of protein [35]. Therefore, we quantified the amount of CSDE1 protein in fetal hypothalamus using Western Blot and observed a significantly higher level of the protein in the PR group in accordance with the level of mRNA expression, but there was no effect due to the sex (Figure 6).

### 3.9. The m6A Epitranscriptomic Mark Was Altered in the PR Fetuses

**Figure 7:**
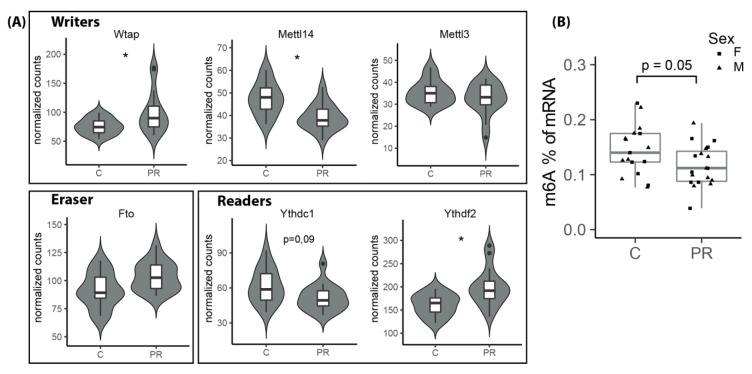
(**A**) Normalized expression level of the genes from the m6A epitranscriptomic machinery obtained using DESeq2 on E17 Hypothalamic RNA sequenced in DGE-seq (PR: *n* = eight males + eight females, C: *n* = eight males + eight females) (adjusted *p*-value from Deseq2 analysis: * padj < 0.05). (**B**) Quantification of the percentage of m6A in total RNA from E17 fetal hypothalamus using m6A immunodetection assay (PR: *n* = 10 males + nine females, C: *n* = 10 males + seven females) (two-way ANOVA group effect *p* = 0.05, sex effect *p* = 0.73) (boxplot: median, first and third quartiles).

The expression of a family of genes involved in the writing/erasing/reading of the m6A epitranscriptomic mark was impacted by maternal PR. The m6A mark is the most abundant modification of mRNA and its involvement in many developmental processes; in particular, neural stem cell fate and neurodevelopment are attracting increasing interest. The *Wtap*, *Mettl14* and *Mettl3* genes encode proteins that constitute the methylation complex in charge of the writing of the m6A mark, whereas the *Fto* and *Alkbh5* encode the erasers that suppress it. The *Mettl14* gene was significantly under-expressed in the PR group (log2FC = −0.27, padj = 0.04), whereas the *Wtap* gene was over-expressed (log2FC = 0.37, padj = 0.03) and the *Mettl3* gene was unchanged (Figure 7A). In addition, the *Fto* gene showed a tendency to be slightly overexpressed in the PR group (log2FC = 0.18, padj = 0.10), and the *Ythdf2* gene, encoding an m6A reader that reduces mRNA stability, was also significantly overexpressed in the PR group (log2FC = 0.29, padj = 0.01) whereas the *Ythdc1* gene, encoding a reader protein involved in mRNA splicing, was slightly under-expressed (log2FC = −0.27, padj = 0.10) in the PR group (Figure 7A).

In relation to the variation in the expression of this family of genes, we next quantified the global level of m6A in the mRNAs from E17 fetal hypothalamus. For that purpose, we used an ELISA-based kit involving an antibody specifically designed against the m6A mark. The percentage of m6A in total mRNA varied from 0.08 to 0.20 and was lower in the PR group (*p* = 0.05), but was not influenced by sex (Figure 7B).

## 4. Discussion

In this study, we used a well-characterized model of maternal protein restriction during gestation to identify gene families and physiological pathways that were altered in the fetal hypothalamus in response to the maternal PR diet.

The impact of an imbalanced maternal diet on the proliferation and differentiation capacities of neural stem cells during embryo and fetal development is now clearly established [18,36,37,38]. Our observations on the cells sampled on E17 fetuses and grown in vitro as neurospheres confirmed these alterations. The total number of cells after dissociation of the fetal hypothalami was lower in the PR group, which may reflect reduced proliferation in an earlier period in the PR group. In addition, we observed, after three days of proliferation in vitro, a higher proportion of TUBB3+ cells in the PR group, which may reflect that E17 PR fetuses had initially a higher number of committed neuronal progenitors that proliferated in culture. Similarly, Gould et al. [18] showed that low protein diet throughout gestation was associated with an increase in the number of late neural progenitors in mice brain but tempered by increased apoptosis. Further investigation would be required in order to establish whether this was also the case in our model.

Interestingly, the proportion of cells expressing NES and MAP2 were not different between groups whereas the genes encoding these proteins were under-expressed in the PR group. This may be related to the fact that the level of expression of these genes varies throughout the differentiation process, from early to late progenitors until differentiated neurons. Therefore, the difference in expression level might reflect an alteration in the timing of differentiation which cannot be seen in the immunochemistry experiments that do not distinguish between cells that have variable levels of gene expression. Additionally, we cannot exclude post-transcriptional control of expression.

E17 corresponds approximately to the time when neurogenesis is complete and residual NPCs start to differentiate into astrocytes [13,14]. Indeed, no cell was GFAP-positive at E17 and the *Gfap* gene was not expressed. The switch between neurogenesis and astrocytogenesis is based upon a complex interaction between external signals and a cell-intrinsic program via a strict control of gene expression. This interaction first requires nutrient sensing and detection of metabolic and hormonal signals coming from the mother and the placenta and then the activation of regulatory pathways that control cell differentiation. By using a large scale transcriptomic approach on whole fetal hypothalamus, we highlighted several metabolic pathways and molecular regulation systems that were impacted by maternal PR and led us to propose some mechanistic hypotheses in order to explain alterations of various neurodevelopment processes.

Our data suggested an alteration of the mitochondrial respiratory chain activity in the PR group, as evidenced both by the over-expression of genes encoding the complexes of the respiratory chain and several enzymes from the pyruvate and citric acid metabolism as well as the increased mitochondrial membrane potential of the E17 hypothalamic cells. Mitochondrial DNA copy number was not different between control and PR fetal hypothalamus, suggesting that the difference in the respiratory activity was not a consequence of a major shift in the number of mitochondria, but possibly a difference in their metabolic activity. One interesting observation that would require to be extended to other proteins from the respiratory chain complexes was the fact that the protein level of four of these proteins was increased mostly in females. Although brain mitochondrial metabolism is known to differ between adult males and females both in human and rodents [39,40], there is, to our knowledge, no data in the literature regarding sex effect on mitochondria dynamics and metabolism in the developing fetal brain. Only the testosterone surge occurring around birth in male mice was shown to impact the synthesis of the mitochondrial-specific phospholipid cardiolipin [41].

Mitochondria dynamics is closely associated with cell fate and differentiation process during brain development. Mitochondria structure and metabolism change throughout the differentiation process [42]. At the metabolic level, while energy production relies mostly on glycolysis in undifferentiated cells, it progressively switches to oxidative phosphorylation throughout neural differentiation in order to meet the higher energy requirements of the differentiated neurons [42]. It has also been illustrated that mitochondria dynamics and metabolic shift precede and functionally regulate neuronal differentiation [43].

Several evidences have already established a link between PR during early life and alterations in the mitochondrial metabolism at a later stage of life. Maternal PR was shown to be associated with (1) impaired mitochondrial metabolism in the brain of adult rat offspring [44] and (2) alteration in the expression level of several proteins from the mitochondrial respiratory chain complexes in the hypothalamus of pre-weaned rat [45]. In addition, in human, mitochondrial metabolism is altered in the placenta of neonates suffering from Intra Uterine Growth Restriction that is often the result of a reduced provision of nutrients to the fetus [46]. Oxidative stress that may results from impaired mitochondrial function is indeed evoked as a major programming mechanism in the increased risk of chronic degenerative diseases induced by neonatal protein restriction [47]. However, although the consequences of neonatal PR on mitochondrial metabolism and oxidative stress on several tissues after birth are widely acknowledged, the link between PR, mitochondrial metabolism in fetal brain and an impaired neurodevelopment is, to our knowledge, not documented. Are mitochondria of neural stem/progenitor cells able to function as nutritional sensors and integrate very early on signals from their environment? In a mice model of maternal protein restriction, Eckert et al. [48] demonstrated that, as early as E3.5, the blastocyst was able to sense maternal metabolic alterations, including deficiency in essential amino acids, within uterine fluid and they showed evidence of the implication of the mammalian Target of Rapamycin Complex 1 (mTORC1) signaling pathway in this process. Mitochondrial activity reflects the energetic status of the cells and mitochondria architecture was recently suggested to play an important role in bioenergetics adaptation to metabolic demands [49]. Therefore, mitochondria dynamics constitute a way for the cell to adapt to nutrient shortage or excess. For instance, nutrient shortage was shown to result in the fusion of mitochondria associated with increased oxidative phosphorylation, which is for the cell the most efficient way to produce ATP [50].

Our data are not sufficient to conclude that mitochondrial respiratory chain activity was definitely increased in the fetal hypothalamus of the PR group and additional experiments are certainly required to confirm this hypothesis. For instance, it could be interesting to measure mitochondrial mass and ROS production. However, the combination of our results regarding this point, together with what is already known about the link between mitochondrial activity and neuronal differentiation strongly supports the impact of maternal PR on these processes.

How the mitochondrial adaptive mechanism, linked to nutrient shortage, interacts with the program of differentiation of neural cells remains to be clarified.

The ubiquitin gene and several genes encoding the subunits of the proteasome complex were also upregulated in the PR fetal hypothalamus. The UPS (Ubiquitin Proteasome System) is closely associated with the mitochondrial metabolism. Mitochondria need the UPS for the removal of proteins that are damaged by ROS (Reactive Oxygen Species) and the proteasome function requires ATP [51]. These two functions are especially important in neuronal function and differentiation [52,53,54] as well as adult neurogenesis [55]. Although the precise mechanisms of the role of the amino acid sensing pathway mTORC1 in the activation of the proteasome activity in situation of nutrient shortage are still under debate [56], these two major pathways are obviously interconnected [57], suggesting that they may interact in the response of neural cells to amino acid shortage. Cellular response to nutrient shortage may also involve autophagy, another major stress-response system that is closely linked to the mTORC1 detection system and which was shown to be important for neuronal development and axon growth [58]. We did not find evidence of alterations in the expression of genes involved in the autophagy in our model but we did notice enrichment in upregulated genes from the lysosome pathway (Table S2).

Epigenetic control of chromatin conformation [59,60], DNA-binding proteins [61] and transcription factors [62,63,64] are among the best known molecular actors of the highly complex process of neuronal differentiation. Their action is itself modulated by factors related to the metabolic status of the cell via the remodeling of chromatin [65]. Recently, post-transcriptional control was highlighted as a new layer of regulation in the determinism of cell fate and differentiation, particularly in the brain [66]. Post transcriptional regulation include (1) chemical modifications of mRNA such as epitranscriptomic marks and (2) RNA-binding proteins that are involved in mRNA stability, turn-over, trafficking, degradation and translation.

We have shown, in our large-scale transcriptomic study, and confirmed (by the quantification of m6A) that the expression of several genes from the m6A epitranscriptomic machinery was significantly disturbed in the PR fetuses and that this was linked to a decrease in the global level of m6A. The m6A epitranscriptomic mark is the major mRNA modification identified so far and is associated with the control of various aspects of mRNA functions including stability, degradation, trafficking, splicing and translation [67]. The brain is the tissue where this mark is the highest and it is especially present in mRNAs implicated in transcriptional regulation, cell adhesion, axon guidance and synaptogenesis [68]. In addition, Yoon et al. [69] recently demonstrated that depleting the writing of the m6A mark by inactivation of the *Mettl14* gene in the embryonic brain of mice prolongs neurogenesis postnatally. This was associated with a decrease in the turnover of mRNAs encoding proteins involved in cell cycle, neurogenesis, and neuronal differentiation. The action of the m6A mark is mediated through interaction with different RNA-binding proteins that specifically recognize methylated or unmethylated mRNAs and will subsequently promote transcript fate [67]. Interestingly, the m6A mark was demonstrated to be involved in the action of the Fragile X Mental Retardation Protein (FMRP) which is an RNA-binding protein with a major role in synapse function [68] and that was shown to be over-expressed in the cortex of mice that suffered from fetal protein restriction [18]. Although the expression of the *Fmr1* gene was not altered in our model, the *Fxr1* gene was downregulated in the PR group. Since this gene also encodes an RNA-binding protein that was recently shown to control the translation of the mitochondrial *Cox2* gene [70] and since *Cox 2* was one of the most expressed and significantly upregulated genes in the PR group, a possible link could exist between the m6A mark, RNA-binding proteins and mitochondrial function. On the other hand, FTO protein, which acts as a m6A demethylase, was shown to have a role in the cellular sensing of amino acids via the mTORC1 pathway and this activity was associated with its demethylase function [71]. In addition, the mTORC1 pathway was demonstrated to mediate the link between nutrient shortage and the control of protein synthesis at the post transcriptional level [66]. Interestingly, the mRNAs that are regulated by this system are enriched for the consensus motif of the m6A epitranscriptomic mark [66]. All these elements converge to propose the m6A mark as a major actor in the impact of amino acid deficiency on hypothalamus development.

The role of post-transcriptional regulation in the consequences of PR on the timing of neurogenesis was also suggested by the over-expression at both the transcription and translation levels of CSDE1 in the PR group. The CSDE1 protein was recently shown to be a master regulator of neuronal differentiation, by regulating, at the translational level, the expression of a large number of genes [35]. The expression of CSDE1 decreases throughout differentiation and modulates the transcriptional landscape by controlling the expression of key regulators of cell fate and neuronal differentiation. Therefore, the overexpression of both the gene and the protein that we observed in the PR group may reflect a delay in the neuronal differentiation process that may be consistent with a higher number of neuronal progenitors.

The DGE-seq approach was rather helpful for the detection of the pathways impacted by maternal diet. Of course, transcriptomic data do not always reflect the amount of proteins, but the DGE-seq approach is rather straightforward and more sensitive that a proteomics approach for the detection of mild effects. The proof is that we have indeed been able to identify key players in post-transcriptional regulation who are certainly involved in the impact of fetal nutrition in the precise control of neuronal differentiation. We made the choice to focus here on metabolic pathways and gene families that were, in our opinion, relevant regarding the physiological and cellular alterations observed in our model, but the transcriptomic approach also identified many other genes that certainly may require further investigation. The magnitude of gene expression change between PR and control fetuses was rather modest. However, major cellular pathways related to energy metabolism and neuronal differentiation have been impacted, so we believe that even mild disturbances can have repercussions on a process as precise and finely regulated as neuronal differentiation.

## 5. Conclusions

In conclusion, our study identified a number of cellular and molecular pathways that could link an environmental event such as PR to alterations in cellular metabolism, leading to early alterations of neuronal development and subsequent impaired hypothalamus function. These hypotheses certainly require further investigation, but we believe that a global approach like ours could be useful in obtaining an overview of all biological systems, including nutrient detection, energy metabolism and the control of transcription/translation, which could link nutritional status to the regulation of cell differentiation, especially in models where the effects are mild and multiple.

## Figures and Tables

**Table 1:** Litter and fetus characteristics at embryonic day 17 (E17).

	Controls		Protein Restricted		*p*-Value
Litter Size	13.00 ± 3.39 (*n* = 13)		14.13 ± 2.61 (*n* = 15)		0.50 *
Maternal Weight Gain (g)	112.39 ± 17.77 (*n* = 13)		99.47 ± 21.12 (*n* = 13)		0.16 *
Fetal Weight (g)	♀ (*n* = 32)	♂ (*n* = 38)	♀ (*n* = 54)	♂ (*n* = 36)	Group: 0.41 ^#^
	0.76 ± 0.05	0.79 ± 0.05	0.77 ± 0.07	0.81 ± 0.06	Sex: <0.0001 ^#^
Placenta Weight (g)	0.39 ± 0.06 (*n* = 70)		0.40 ± 0.07 (*n* = 81)		0.85 *
PFR (Placenta–Fetus Ratio)	0.51 ± 0.09 (*n* = 70)		0.52 ± 0.10 (*n* = 81)		0.83 *

* Mann–Whitney test; ^#^ two-way ANOVA test.

## Supplementary Materials

The following are available online at https://www.mdpi.com/2072-6643/12/5/1464/s1. Table S1: DESeq2 output [27] obtained from the differential gene expression analysis. BaseMean column indicates mean of normalized counts for each gene at all ages used for DGE-seq (E17, D0 and D130). BaseMeanE17 column indicates mean of normalized counts for each gene only at E17. Padj: adjusted *p*-value. DE: Differential Expression (UP: PR > C padj < 0.05; DOWN: PR < C padj < 0.05; NONE: padj > 0.05). Table S2: FGSEA (Fast Gene Set Enrichment Analysis) output [29] obtained from functional enrichment analysis using Reactome, KEGG and GO databases.

## Appendix A

**Table A1:** Top 20 upregulated genes in the protein-restricted group.

Gene Symbol	Log2FC	Padj	Base Mean	Full Name	Function(s) *
*Slc35c2*	0.469	0.031	24.122	Solute carrier family 35 member C2	May play a role in cellular response to tissue hypoxia
*Ppcs*	0.463	0.042	12.650	Phosphopantothenoylcysteine synthetase	Involved in the biosynthesis of coenzyme A, a precursor of Acetyl CoA
*Csde1*	0.452	0.001	817.685	Cold shock domain containing E1	RNA-binding protein—prevents neuronal differentiation in neural stem cells
*Nipa2*	0.437	0.028	19.597	Nipa magnesium transporter 2	Non-imprinted gene in Prader–Willi/Angelman syndrome region
*Coa5*	0.429	0.006	117.035	Cytochrome C oxydase assembly factor 5	Involved in the mitochondrial complex IV assembly
*Cirbp*	0.428	0.002	250.612	Cold inducible RNA-binding protein	Essential for embryonic gastrulation and neural development
*Hs6st1*	0.423	0.030	91.038	Heparan-sulfate 6-O-sulfotransferase 1	Critical for normal neuronal development, may play a role in neuron branching
LOC100174910	0.418	0.010	48.852	Glutaredoxin-like protein	Involved in oxidation-reduction process. May be involved in cell redox homeostasis
*Wdr83*	0.407	0.044	34.218	WD repeat domain-containing protein 83	Involved in response to hypoxia
*Tmem53*	0.406	0.025	27.565	Transmembrane protein 53	
*Ube2n*	0.405	0.002	83.937	Ubiquitin conjugating enzyme E2N	Involved in protein ubiquitination. Plays a role in the control of cell cycle and differentiation
*Tmcc2*	0.397	0.044	55.090	Transmembrane and coiled-coil domains protein 2	Expressed in endoplasmic reticulum
*Sec23b*	0.395	0.044	23.013	COPII coat complex component	Involved in protein transport from endoplasmic reticulum
*Coq7*	0.393	0.001	120.626	coenzyme Q7 hydroxylase	Involved in ubiquinone synthesis, in mitochondrial respiratory metabolism
*Chrac1*	0.391	0.006	66.458	Chromatin accessibility complex protein 1	Histone-fold protein that binds DNA. May be involved in cell growth and survival
*Rbm7*	0.377	0.044	20.552	RNA-binding protein 7	Member of the exosome targeting complex, involved in RNA degradation
*Wtap*	0.373	0.031	87.528	WT1 associated protein	Member of the complex that mediate m6A methylation of RNAs
*Mterf1*	0.367	0.032	30.933	Mitochondrial transcription termination factor 1	DNA-binding protein, involved in termination of mitochondrial transcription
*Marcks*	0.365	0.026	1055.600	Myristoylated alanine rich protein kinase C substrate	Binds protein of cytoskeleton, may be involved in cell migration
*Pqlc1*	0.365	0.044	39.620	Solute carrier family 66 member 2	Involved in phospholipid translocation

*: from the UNIPROT database (www.uniprot.org) [72] and the NCBI/Gene database (www.ncbi.nlm.nih.gov/gene) [73].

**Table A2:** Top 20 downregulated genes in the protein-restricted group.

Gene Symbol	log2FC	Padj	Base Mean	Full Name	Function(s) *
*Brsk1*	−0.807	0.010	13.066	Serine Threonine protein kinase	Role in polarization of neurons and centrosome duplication
*Fgfr1op*	−0.676	0.002	19.199	FGFR1 oncogen partner	Required for anchoring microtubules to the centrosomes. Involved in cell proliferation
*Qk*	−0.658	0.003	22.941	Quaking	RNA-binding protein that regulates pre-mRNA splicing, export, stability and translation. Involved in oligodendrogenesis
*Tcf20*	−0.636	0.024	19.373	Transcription factor 20	Transcriptional activator. May be involved in neurodevelopment
*Cask*	−0.625	0.013	23.749	Calcium/calmodulin dependent serine protein kinase	Involved in synaptic membrane protein anchoring. Contributes to neurodevelopment
*Eps15*	−0.607	0.021	12.515	Epidermal growth factor receptor pathway substrate 15	Involved in cell growth regulation. May be involved in the control of cell proliferation
*Robo1*	−0.600	0.009	56.779	Roundabout guidance receptor 1	Involved in axon guidance and neuronal precursor cell migration
*Fam168a*	−0.569	0.010	19.145	Also known as Tcrp1	Involved in cancer chemotherapy resistance
*Iqgap1*	−0.566	0.009	18.290	IQ motif containing GTPase activating protein 1	Regulates the assembly and dynamic if the cytoskeleton. May promote neurite outgrowth.
*Phactr3*	−0.560	0.025	15.498	Phosphatase and actin regulator 3	Associated with the nuclear scaffold in proliferating cells
*Myo18a*	−0.548	0.009	28.595	Myosin 18A	Associated with the Golgi. May be required for cell migration
*Sesn3*	−0.541	0.045	17.734	Sestrin 3	Required for regulation of glucose and insulin regulation. May be involved in the protection againt oxidative stress
*Kcnma1*	−0.530	0.044	16.245	Potassium calcium-activated channel subfamily M alpha 1	Involved in the control of neuron excitability
*Pclo*	−0.529	0.002	95.164	Piccolo presynaptic cytomatrix protein	Involved in synaptogenesis
*Mgat3*	−0.526	0.024	19.383	beta−1,4-mannosyl-glycoprotein 4 beta-N-Acetylglucosaminyltransferase	May be involved in response to oxidative stress in brain and in cell migration regulation
*Timp3*	−0.526	0.049	10.279	TIMP metallopeptidase inhibitor 3	
*Taok1*	−0.518	0.007	43.989	Serine Threonine protein kinase TAO1	Acts as a regulator of cytoskeleton stability. May be involved in the induction of apoptosis
*Nlgn1*	−0.515	0.031	15.813	Neuroligin 1	Neuronal cell surface protein. May be involved in the formation and remodeling of synapses
*Kpnb1*	−0.511	0.025	18.776	Karyopherin subunit beta 1	Involved in nuclear protein import, including ribosomal proteins and histone H1
*Slmap*	−0.510	0.028	14.084	Sarcolemma associated protein	Membrane associated protein

*: from the UNIPROT database (www.uniprot.org) [72] and the NCBI/Gene database (www.ncbi.nlm.nih.gov/gene) [73].
